# Supplementary material for: Is Segmental Ureterectomy Associated with Inferior Survival for Localized Upper-Tract Urothelial Carcinoma of the Ureter Compared to Radical Nephroureterectomy?
Source: Cancers (Basel). 2023 Feb 21;15(5):1373. doi: 10.3390/cancers15051373 (PMC10000204; doi:10.3390/cancers15051373)
Supplement: Supplementary file 1 [file cancers-15-01373-s001.zip › cancers-2168234-supplementary.pdf]

Table S1. Comparison of the baseline characteristics of patients affected by urothelial carcinoma of the ureter who were included in the study vs those who were excluded.

Characteristic		Excluded patients N = 11,049 (45.8%)	Included patients N = 13,061 (54.2%)	*p*-value
Age categories, years	< 60	1146 (10.4)	1559 (11.9)	>0.001
	60–69	2497 (22.6)	3441 (26.4)	
	70–79	3749 (33.9)	4852 (37.2)	
	> 80	3657 (33.1)	3209 (24.6)	
Gender, N (%)	Male	6661 (60.3)	8239 (63.1)	>0.001
	Female	4388 (39.7)	4822 (36.9)	
Race, N (%)	White	10,179 (92.1)	12,104 (92.7)	0.125
	Black	427 (3.9)	443 (3.4)	
	Other	349 (3.2)	399 (3.1)	
	Unknown	94 (0.9)	115 (0.9)	
Comorbidity index, N (%)	0	7433 (67.3)	8377 (64.1)	>0.001
	1	2502 (22.6)	3281 (25.1)	
	2	804 (7.3)	1028 (7.9)	
	≥3	310 (2.8)	375 (2.9)	
Insurance status, N (%)	Private	2392 (21.7)	3218 (24.6)	>0.001
	Medicaid	360 (3.3)	364 (2.8)	
	Medicare	7895 (71.5)	9170 (70.2)	
	Uninsured	137 (1.2)	146 (1.1)	
	Unknown	265 (2.4)	163 (1.3)	
Income, N (%)	High	6817 (61.7)	8054 (61.7)	0.223
	Low	4186 (37.9)	4932 (37.8)	
	Unknown	46 (0.4)	75 (0.6)	
Education, N (%)	High	6651 (60.2)	8103 (62.0)	0.002
	Low	4358 (39.4)	4891 (37.5)	
	Unknown	40 (0.4)	67 (0.5)	
Facility type, N (%)	Academic	3959 (35.8)	4563 (34.9)	0.064
	Non-academic	7050 (63.8)	8467 (64.8)	
	Unknown	40 (0.4)	31 (0.2)	
Facility location, N (%)	East	4839 (44.3)	5766 (44.2)	>0.001
	Central	4383 (39.7)	5546 (42.5)	
	West	1733 (15.7)	1718 (13.2)	
	Unknown	40 (0.4)	31 (0.2)	
Facility county (%)	Metro	8928 (80.8)	10,531 (80.6)	0.805
	Urban	1626 (14.7)	1928 (14.8)	
	Rural	197 (1.8)	255 (2.0)	
	Unknown	298 (2.7)	347 (2.7)	
Facility distance (%)	First	6318 (57.2)	7158 (54.8)	>0.001
	Second	3228 (29.2)	4194 (32.1)	
	Third	1470 (13.3)	1653 (12.7)	
	Unknown	33 (0.3)	56 (0.4)	
Clinical T stage (%)	≤T1	4648 (42.1)	5158 (39.5)	>0.001
	T2	629 (5.7)	1079 (8.3)	
	T3	656 (5.9)	827 (6.3)	
	T4	422 (3.8)	92 (0.7)	
	Unknown	4694 (42.5)	5905 (45.2)	

Tumor size (%)	<= 2 cm	2064 (18.7)	3675 (28.1)	>0.001
	> 2 cm	3155 (28.6)	6505 (49.8)	
	Unknown	5830 (52.8)	2881 (22.1)	
Tumor grade (%)	Low grade	2,040 (18.5)	1975 (15.1)	>0.001
	High grade	3,178 (28.8)	4392 (33.6)	
	Unknown	5831 (52.8)	6694 (51.3)	
Lymph vascular invasion (%)	Not present	2867 (26.0)	4488 (34.4)	>0.001
	Present	548 (5.0)	954 (7.3)	
	Unknown	7634 (69.1)	7619 (58.3)	
Chemotherapy (%)	Not received	10,504 (78.5)	10,997 (84.2)	>0.001
	Received	1956 (17.7)	1568 (12.0)	
	Unknown	422 (3.8)	496 (3.8)	

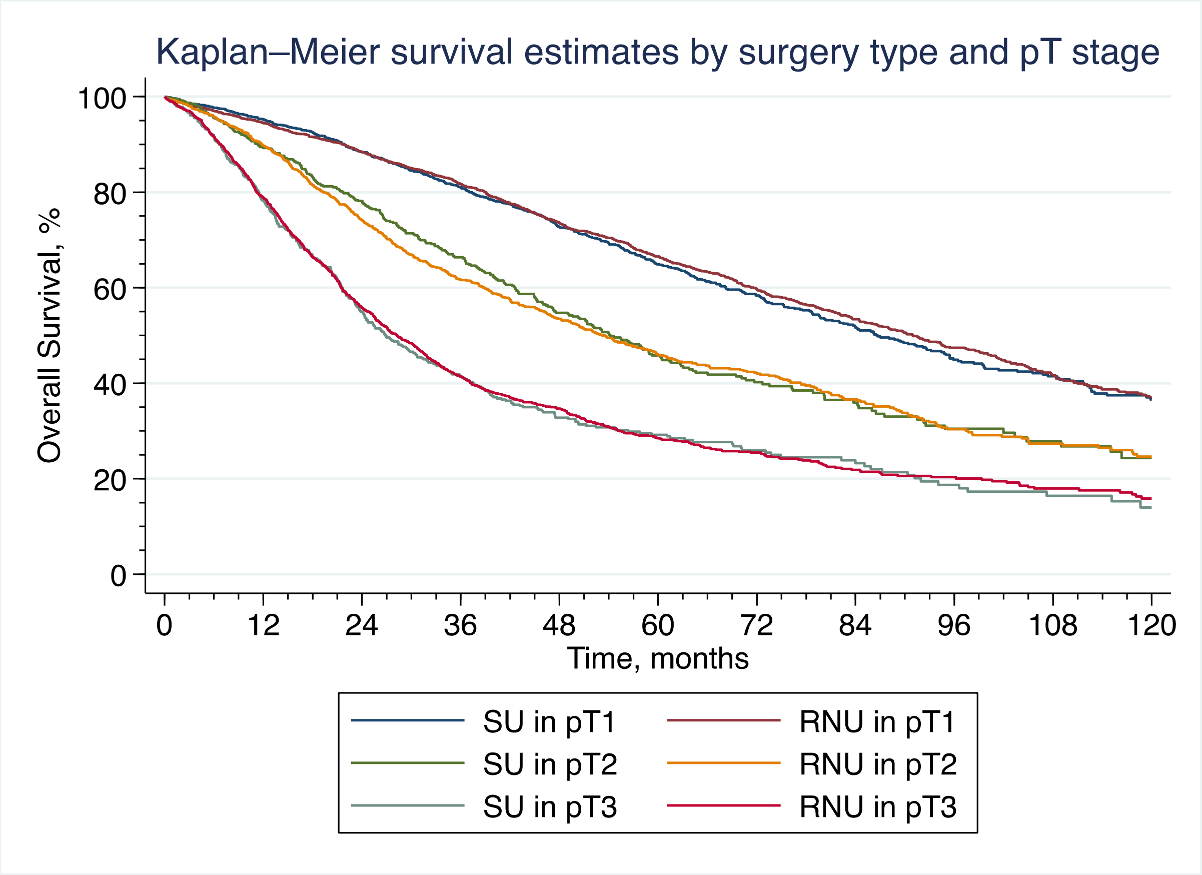

FigureS1. Overlap weighting-adjusted Kaplan-Meier analysis of overall survival in patients who received Radical Nephroureterectomy vs Segmental Ureterectomy, according to pathological T stage. SU= Segmental Ureterectomy, RNU= Radical Nephroureterectomy, pT stage= pathological T stage.
